# Integrative computational analysis of transcriptional and epigenetic alterations implicates *DTX1* as a putative tumor suppressor gene in HNSCC

**DOI:** 10.18632/oncotarget.14856

**Published:** 2017-01-27

**Authors:** Daria A. Gaykalova, Veronika Zizkova, Theresa Guo, Ilse Tiscareno, Yingying Wei, Rajita Vatapalli, Patrick T. Hennessey, Julie Ahn, Ludmila Danilova, Zubair Khan, Justin A. Bishop, J. Silvio Gutkind, Wayne M. Koch, William H. Westra, Elana J. Fertig, Michael F. Ochs, Joseph A. Califano

**Affiliations:** ^1^ Department of Otolaryngology—Head and Neck Surgery, Johns Hopkins Medical Institutions, Baltimore, Maryland, USA; ^2^ Institute of Molecular and Translational Medicine, Faculty of Medicine and Dentistry, Palacky University, Olomouc, Czech Republic; ^3^ Division of Oncology Biostatistics, Department of Oncology, Johns Hopkins Medical Institutions, Baltimore, Maryland, USA; ^4^ Department of Urology, Northwestern University, Chicago, Illinois, USA; ^5^ Department of Otolaryngology, Mid-Michigan Ear Nose and Throat, East Lansing, Michigan, USA; ^6^ Department of Pathology, Johns Hopkins Medical Institutions, Baltimore, Maryland, USA; ^7^ Department of Pharmacology, UC San Diego Moores Cancer Center, La Jolla, California, USA; ^8^ Department of Mathematics and Statistics, The College of New Jersey, Ewing, New Jersey, USA; ^9^ Department of Surgery, UC San Diego, Moores Cancer Center, La Jolla, California, USA; ^10^ Department of Statistics, The Chinese University of Hong Kong, NT, Shatin, Hong Kong; ^11^ Laboratory of Systems Biology and Computational Genetics, Vavilov Institute of General Genetics, Russian Academy of Sciences, Moscow, Russia

**Keywords:** HNSCC, DTX1, expression, methylation, integration

## Abstract

Over a half million new cases of Head and Neck Squamous Cell Carcinoma (HNSCC) are diagnosed annually worldwide, however, 5 year overall survival is only 50% for HNSCC patients. Recently, high throughput technologies have accelerated the genome-wide characterization of HNSCC. However, comprehensive pipelines with statistical algorithms that account for HNSCC biology and perform independent confirmatory and functional validation of candidates are needed to identify the most biologically relevant genes. We applied outlier statistics to high throughput gene expression data, and identified 76 top-scoring candidates with significant differential expression in tumors compared to normal tissues. We identified 15 epigenetically regulated candidates by focusing on a subset of the genes with a negative correlation between gene expression and promoter methylation. Differential expression and methylation of 3 selected candidates (*BANK1*, *BIN2*, and *DTX1*) were confirmed in an independent HNSCC cohorts from Johns Hopkins and TCGA (The Cancer Genome Atlas). We further performed functional evaluation of NOTCH regulator, *DTX1*, which was downregulated by promoter hypermethylation in tumors, and demonstrated that decreased expression of *DTX1* in HNSCC tumors maybe associated with NOTCH pathway activation and increased migration potential.

## INTRODUCTION

As the fifth most common cancer, Head and Neck Squamous Cell Carcinoma (HNSCC) is responsible for 600,000 new cases and over 300,000 deaths per year worldwide [1, 2]. Nonetheless, the majority of HNSCC patients are diagnosed at an advanced stage due to the asymptomatic course of early stage disease and the absence of the routine screening techniques [3–6]. Development of low toxicity targeted therapeutics and biomarkers for early detection could improve survival rate and quality of life for HNSCC patients.

Traditionally, research groups have focused on the roles of individual genes in HNSCC to develop candidate biomarkers for diagnosis and treatment selection [7]. The process of single-gene investigation is time and labor intensive, while high-throughput profiling enables more rapid discovery of targetable disease-specific modifications. Thus, recent high throughput profiling of HNSCC identified number of targetable disease-specific modifications, such as genetic mutations and differentially expressed genes in HNSCC primary tissues or cell lines compared to normal samples [8–16]. In addition, epigenetic based expression alterations are noted to drive key biologic processes in HNSCC [17, 18]. Integration of high throughput data from expression and methylation platforms may enhance accurate discovery of cancer-driving genes so they can be used as disease biomarkers or therapy targets [19].

Therefore, a multi-platform high throughput analyses of gene expression and DNA methylation in primary HNSCC and normal samples and outlier statistics [20] were utilized to rank candidate genes and prioritize genes with the most prominent abnormalities in tumor samples that were absent in normal samples. Next, candidate genes were validated in separate clinical cohorts. Finally, the functional role of a lead candidate, *DTX1*, in HNSCC cell migration was demonstrated. *DTX1* expression was found to be increased in samples with decreased NOTCH pathway activity, suggesting that *DTX1* can serve as a biomarker of NOTCH pathway inhibition. The promoter DNA was exclusively methylated in tumors, suggesting that it can also serve as a HNSCC biomarker. This discovery was made possible due to employment of well-considered statistical approaches, cross cohort validation, and complementary detection tools.

## RESULTS

### Candidate genes with differential expression in HNSCC detected by outlier statistics

In order to identify relevant gene candidates in HNSCC, we used gene expression array data from a discovery cohort of 44 HNSCC primary tumors and 25 non-cancer normal tissue samples described in our previous publications (Supplementary Table 1 and [21–23]). Notably, the clinical differences between tumor and control population of the discovery cohort were identified and discussed earlier [21–24].

**Figure 1:**
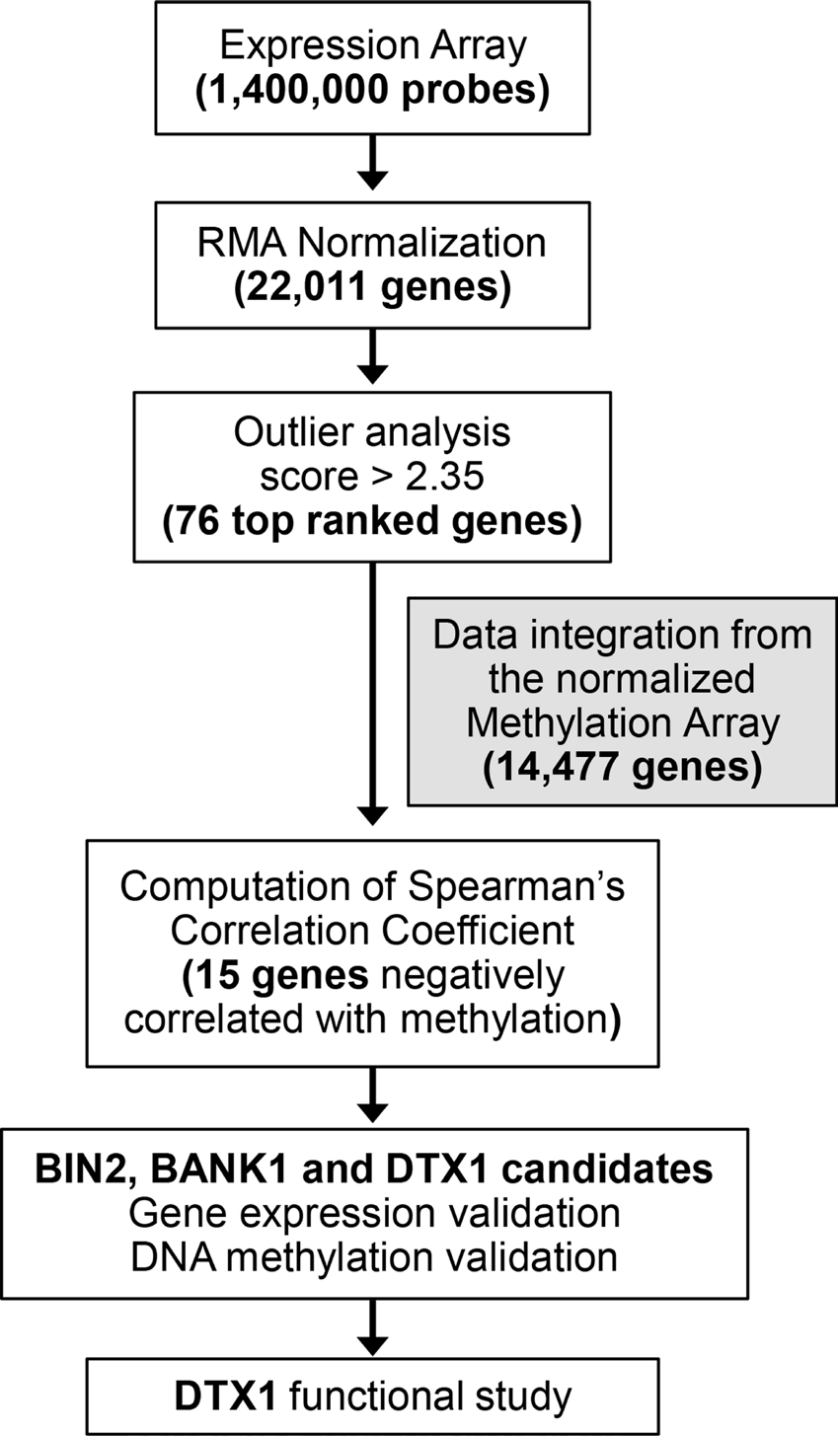
Experimental flow Expression array probes, 1.4M total, were normalized using RMA package. Gene level estimates were produced by choosing the highest mean expression levels among all probes linked to the same gene for expression, yielding 22,011 genes. We applied outlier analysis [20] to the gene expression data set, containing 22,011 genes. The outlier score cut-off for expression data was set at 2.3, resulting in prioritizing 76 top scoring expression candidates. Spearman gene expression-methylation for these 76 candidates was calculated via integration of normalized methylation array data available for the samples. Fifteen out of 76 candidates were found to have a negative Spearman coefficient. Differential expression and methylation of *BIN2*, *BANK1* and *DTX1* were validated in the validation and TCGA-HNSCC cohorts. Functional study was performed for *DTX1*.

The novelty of the current study was that we applied outlier analysis adopted from Ochs et al. [20] to rank and prioritize cancer-related alterations in HNSCC samples relative to normal controls (Figure 1). Outlier analysis is adapted for the study of heterogeneous samples, such as HNSCC primary tissues, because it is sensitive to alterations that maybe present in only a subset of samples. Given the high sensitivity of whole-genome gene expression analysis, thousands of differentially expressed genes can be detected while comparing tumor and normal samples. The heterogeneity of genetic and epigenetic alterations in solid tumors has presented challenges in using conventional statistical approaches, such as *t*-tests or signal-to-noise tests. There are several most-commonly accepted methods employed in cancer research for analysis of high-throughput data of heterogeneous cancers, including Cancer Outlier Profile Analysis (COPA)-based methodology, which compares outliers to an empirical null [25–28]. Outlier-based analysis has provided a mechanism to define significant, but diverse, alterations in cancers [20]. To eliminate low-signal outliers, this work implemented COPA-based statistics with a rank sum outlier approach as well as set a minimum level for the calling of an outlier. Such outlier analysis was recently successfully implemented and validated in a wet-lab setting for the discovery of tumor-specific signatures from DNA methylation array data for HNSCC [29].

Based on the number of outlier samples and the relative signal intensity, 76 of the top ranking candidates were chosen for further analysis (Supplementary Table 2). Overall, 50 candidate genes demonstrated increased gene expression and 26 candidate genes demonstrated decreased gene expression in tumor samples. (Supplementary Table 2). Notably, the standard and more stringent *t-test* demonstrated that 70 out of 76 genes (92%) had statistically significant difference in gene expression between normal and tumor samples.

### Negative correlation between expression and methylation identifies candidate genes that are epigenetically silenced in HNSCC

High gene promoter methylation if often associates with decreased gene expression and can result in epigenetic silencing [17, 18]. Therefore, DNA methylation array data was integrated with gene expression analysis (Figure 1) to identify gene expression changes potentially driven by methylation, as well as to eliminate biases from individual high-throughput platform [23]. The Illumina 27 DNA methylation array was utilized, which contained 27,000 probes covering approximately 15,000 genes, including gene promoter probes for 19 of the 76 candidates. Spearman correlation coefficients were calculated (Supplementary Table 2), and 15 out of 19 genes had negative correlation between expression and methylation. The description of these 15 candidates can be found in Table 1.

**Table 1:** Fifteen candidate genes with negative expression-methylation correlation

#	Gene	Description	Expression-Methylation Correlation, Spearman coefficient	Outlier score	Alteration in different tumor types	Reference
1	*ATP2A3*	ATPase	−0.120	2.45	HNSCC, lung, colon, cancers of central nervous system	[50]
2	*ATP8A1*	ATPase	−0.195	2.60	Lung cancer	[73]
3	*BANK1*	Scaffold protein	−0.420	4.97	Lymphoma, colorectal cancer	[30, 32]
4	*BIN2*	Bridging integrator	−0.693	2.94	myeloproliferative neoplasm	[31]
5	*CD79B*	immunoglobulin-beta protein	−0.556	3.16	Myeloma, CLL	[74, 75]
6	*CYP1B1*	Cytochrome	−0.183	2.50	Smoking related cancers, ovarian cancer	[51, 52]
7	*DTX1*	Notch-pathway regulator	−0.274	3.40	thymic tumor, glioblastoma, osteoblastoma	[46, 47]
8	*FZD3*	Frizzled receptor	−0.161	3.02	Colorectal, non−melanoma skin cancer, CLL	[53−55]
9	*GRAP*	cytoplasmic signaling protein	−0.444	2.93	medullary thyroid carcinoma	[34]
10	*INA*	Neurofilament	−0.333	2.69	colorectal cancer, adenomas	[35]
11	*MAP4K1*	MAP kinase	−0.590	2.58	Bladder, colorectal cancer	[36, 38]
12	*ORAOV1*	Oral cavity oncogene	−0.064	4.00	oral SCC	[56]
13	*PDE5A*	phosphodiesterase	−0.10	3.51	melanoma	[76]
14	*TNFRSF13C*	TNF receptor	−0.295	3.12	non-Hodgkin lymphoma	[37]
15	*VAV1*	proto-oncogene, a member of guanine nucleotide exchange factors	−0.208	2.64	neuroblastoma, lung, pancreatic cancer	[77, 78]

Genes are in alphabetic order. Spearman coefficient and Outlier score are provided.

**Figure 2:**
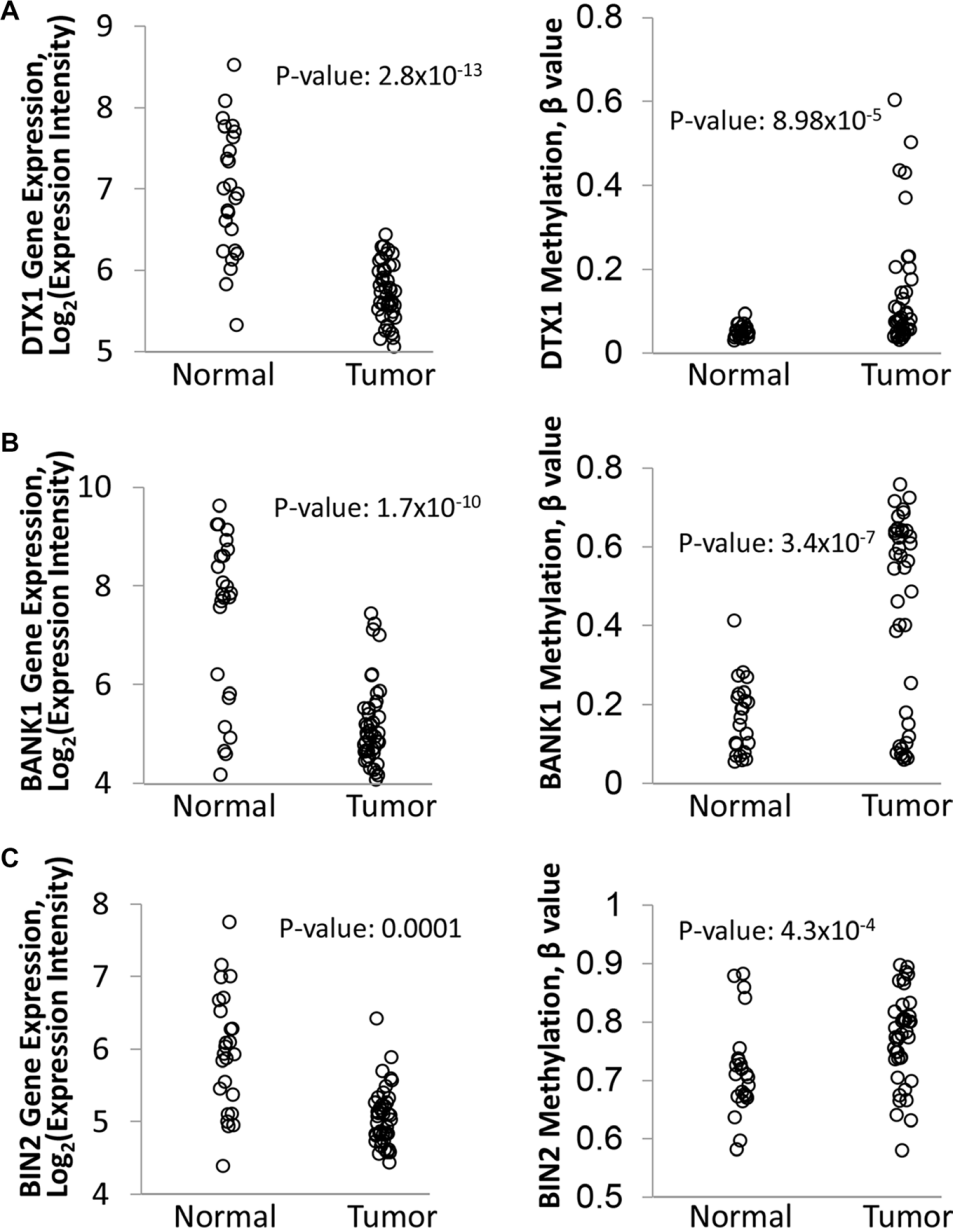
Differential expression and methylation of *DTX1* (**A**), *BANK1* (**B**) and *BIN2* (**C**) in the original discovery cohort. Gene expression (left) was evaluated by Affimetrix HuEx1.0 GeneChip. DNA methylation (right) was evaluated by Illumina Infinium HumanMethylation27 BeadChip platform, and the data was normalized and processed as described in methods. *P-value* were calculated by *t-test*.

Of the list of 15 candidates, *BANK1* (a scaffold protein) and *BIN2* (a bridging integrator protein) had the highest combined outlier score and negative Spearman coefficient. *DTX1* was chosen for its relatively high outlier score and its regulatory role in the NOTCH pathway, which is commonly dysregulated in HNSCC [12–14, 22]. All 3 genes have been implicated as potential cancer drivers in other non-head and neck solid tumors [30–33]. Notably, all 3 genes were downregulated and hypermethylated in tumor samples compared to normal controls (Figure 2). Therefore these 3 genes were selected for further validation. Other candidates, such as *CD79B*, *MAP4K1*, *GRAP*, *TNFRSF13C*, and *INA*, had comparable scores and are candidates for further study, as they have also been implicated in carcinogenesis of other cancer types [34–38].

### Independent validation of differential expression and methylation of *BANK1*, *BIN2* and *DTX1*

**Figure 3:**
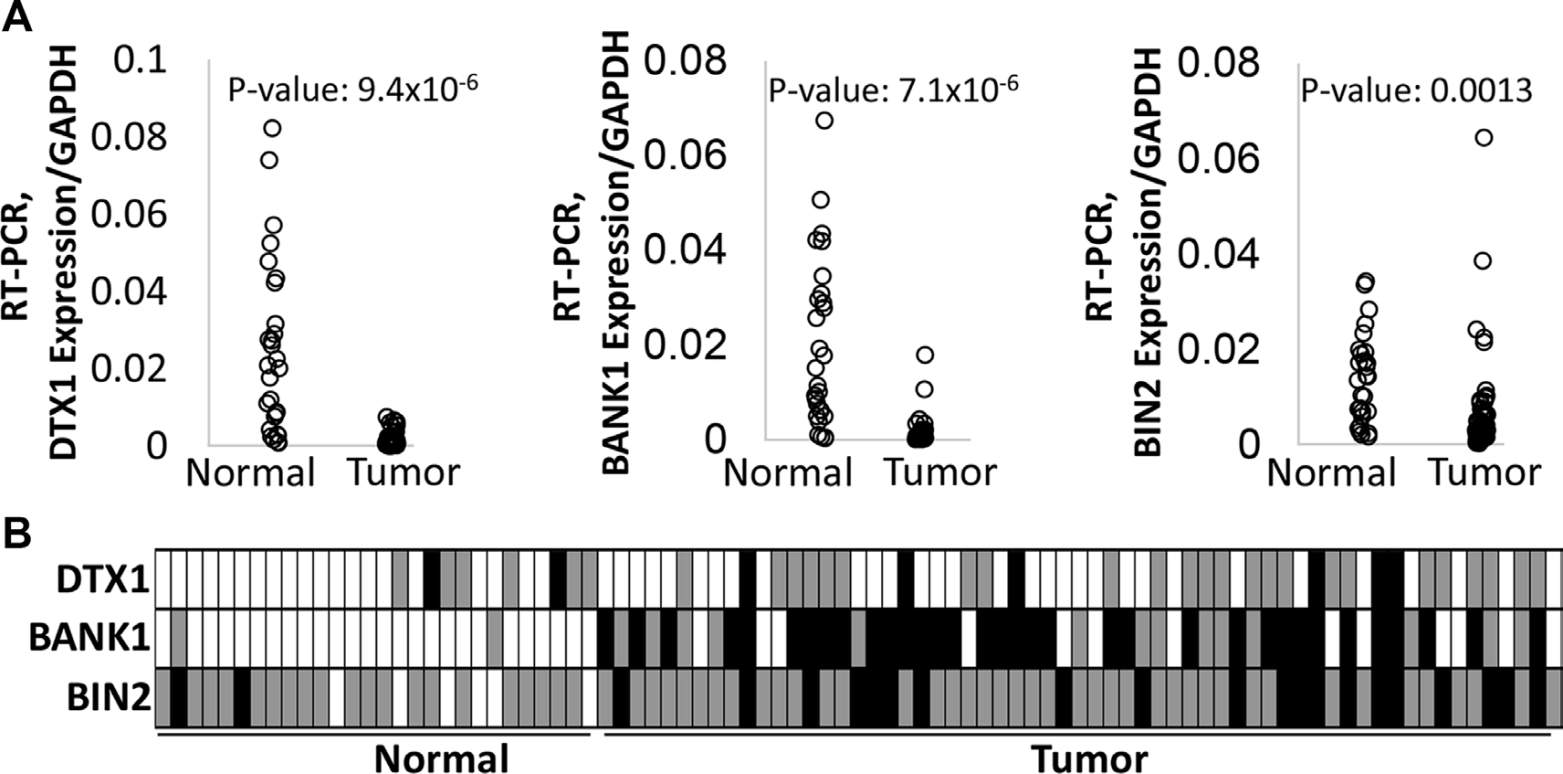
Differential expression and methylation of *DTX1*, *BANK1* and *BIN2* in the validation cohort Gene expression (**A**) was evaluated by quantitative RT-PCR. *P*-values were calculated by *t-test*. DNA methylation (**B**) was evaluated by bisulfite sequencing. Box color-code: white–unmethylated (hypomethylated); grey–hemimethylated, black–hypermethylated. *P*-values were calculated by Fisher exact test, as unmethylated signal vs methylated signal (hemi- or hypermethylated) in two groups. *P*-values for *DTX1* = 0.105, for *BANK1* < 0.0001, for *BIN1* = 0.0006.

To confirm the differential expression and methylation of *BANK1*, *BIN2* and *DTX1*, an independent validation cohort was assembled of 61 HNSCC primary tumors and 28 UPPP samples, with similar clinical characteristics as the discovery cohort (Supplementary Table 1). *BANK1*, *BIN2* and *DTX1* gene expression was evaluated by qRT-PCR, and DNA methylation was evaluated by bisulfite sequencing (Figure 3, Supplementary Table 3). Gene expression was significantly decreased in tumor tissues for all 3 genes (*t-test* *p*-values: 9.4 × 10^−6^, 7.1 × 10^−6^, and 0.0013 for *DTX1, BANK1*, and *BIN2*, respectively), and DNA methylation was present in significantly more tumor samples (Fisher exact test *p*-values: 0.105, < 0.0001, and 0.0006 for *DTX1, BANK1*, and *BIN2*, respectively). Utilization of the independent cohort of HNSCC samples analyzed by complementary methodology (Figure 3) enhanced the rigor of the data through technical validation and eliminated potential sample biases. Notably, for *DTX1* differential methylation did not reach statistical significance (*p* = 0.105). However, *DTX1* showed almost no methylation in normal tissues, resulting in high tumor specificity. Therefore, *DTX1* was maintained as a gene candidate for further study.

**Figure 4:**
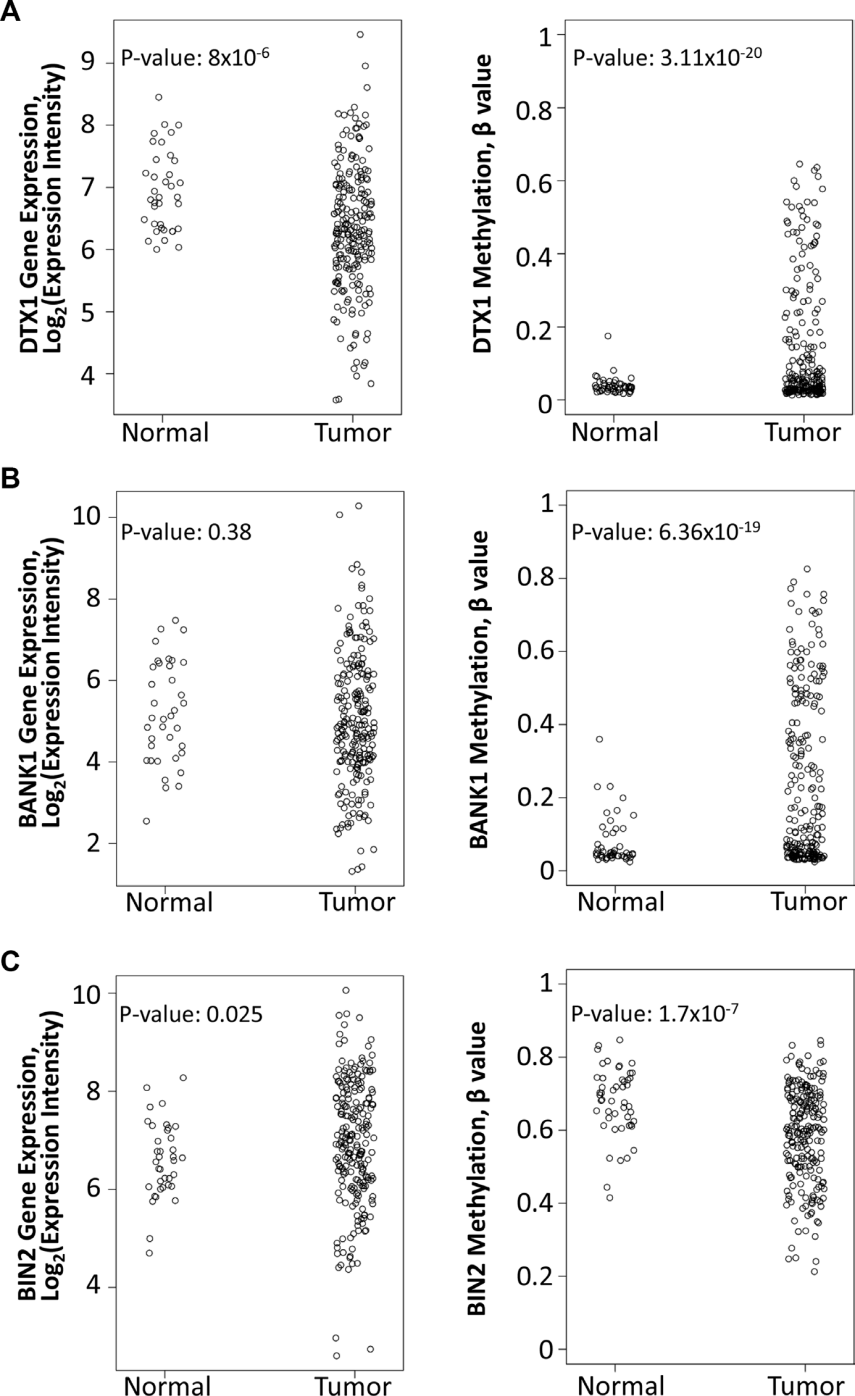
Differential expression and methylation of *DTX1* (**A**), *BANK1* (**B**) and *BIN2* (**C**) in the TCGA-HNSCC cohort. Gene expression (left) was evaluated by RNA-Seq. DNA methylation (right) was evaluated by Illumina Infinium HumanMethylation450 BeadChip platform, and the data was normalized and processed as described in methods. *P*-values were calculated by *t-test*.

High throughput gene expression and DNA methylation analysis was also recently performed by TCGA (The Cancer Genome Atlas), including 222 matched HNSCC tumors and 50 normal samples (Supplementary Table 1 and [39]). TCGA used RNA-Seq for gene expression evaluation and Illumina Infinium HumanMethylation450 BeadChip platform for DNA methylation analysis. We used the TCGA dataset for *BANK1*, *BIN2* and *DTX1* validation (Figure 4). TCGA was not used for initial discovery because use of adjacent normal tissue from cancer patients was a concern. In the HNSCC population, high rate of tobacco and alcohol exposure lead to field of cancerization effect, as well as genetic and epigenetic changes can be seen in tumor adjacent apparently normal tissue [40, 41].

Nonetheless, data from TCGA was able to provide additional independent validation. Within TCGA, *DTX1* was found to have significantly decreased expression (*p-value* = 8 × 10^-6^) and promoter hypermethylation (*p-value* = 3.11 × 10^−20^), validating prior results. However *BANK1* shared promoter hypermethylation (*p-value* = 6.36 × 10^-19^) without significant changes in gene expression (*p-value* = 0.38). For *BIN2* neither gene expression downregulation nor hypermethylation changes were validated in TCGA. Since *DTX1* showed the best performance in TCGA cross validation, we focused our further functional validation only on the *DTX1* gene.

### The role of DTX1 in the NOTCH pathway for HNSCC

DTX1 is a regulator of the NOTCH pathway [42, 43]. Since the NOTCH pathway is dysregulated in HNSCC [12, 13, 22], we evaluated the role of DTX1 relative to other NOTCH pathway genes (KEGG database and [22]). Overall, we analyzed gene expression for 44 NOTCH related genes for 44 HNSCC and 25 normal controls from the discovery cohort using Affymetrix Exon Array data (Supplementary Figure 1). Genes were sorted by unsupervised hierarchical clustering. *DTX1* was noted to cluster together with *DTX3* (NOTCH regulator), *MFNG* (NOTCH modifier) and *DLL3* (NOTCH ligand).

Interestingly, separation of HNSCC samples by *DTX1* expression separated the tumor samples into two subsets of samples, with different expression of NOTCH pathway genes in each group (Supplementary Figure 1). To confirm this observation, we separated 44 tumors into two equal groups by *DTX1* expression: lower *DTX1* expression (*n* = 22) and higher *DTX1* expression (*n* = 22) and compared the expression of NOTCH genes in each group (Supplementary Table 4). Indeed, 34 out of 43 NOTCH pathway genes excluding *DTX1* (79%) were significantly differentially expressed between *DTX1* low and high expressed tumors. According to Supplementary Figure 1 and Supplementary Table 4, the majority of NOTCH pathway genes were significantly upregulated in the group of samples with lower *DTX1* expression (*n* = 28 genes, including *NOTCH1-3*, *HES1*, *HEY1*, and *JAG1*) relative to *DTX1* high expression samples. Fifteen genes had lower expression in the *DTX1* lower-expression group relative to *DTX1* high expression samples, including *DLLs*, *DTXs* and *NOTCH4* genes. Gene set analysis confirmed that the set of NOTCH pathway genes were significantly overexpressed in *DTX1* low samples relative to *DTX1* high (*p-value* of 0.0012, Supplementary Figure 2) and relative to Normal samples (*p-value* of 0.0024, Supplementary Figure 2). These findings confirm that downregulation of *DTX1* results in a strong difference in activation of the NOTCH pathway in HNSCC samples.

We further evaluated relative expression of *DTX1* with NOTCH downstream targets (Supplementary Figure 3). *DTX1* expression co-clustered with the expression of *GATA4* (regulated by *HEY1*) [44] and *NEUROG3* (negatively regulated by *HES1*) [45] by unsupervised hierarchical clustering. The set of NOTCH downstream targets were not significantly differentially expressed between *DTX1* sample groups or relative to normal samples with gene set analysis. Nonetheless, *GATA4* and *NEUROG3* were all downregulated in samples with low *DTX1* expression relative to high *DTX1* expression (*p*-values of 7 × 10^−7^ and 6 × 10^−10^, respectively; Supplementary Table 5). In addition, *DTX1* low samples had significantly higher expression of *HES1* relative to *DTX1* high samples (*p-value* of 0.01; Supplementary Table 5), consistent with *NEUROG3* downregulation [45], but significant changes were not observed from gene set statistics (data not shown).

### Functional role of DTX1 dysregulation in HNSCC

**Figure 5:**
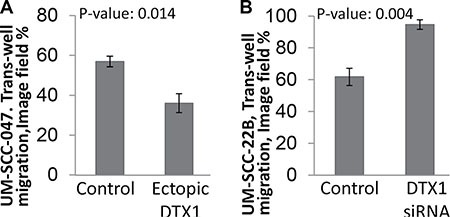
*DTX1* blocks HNSCC invasiveness Migration assay was performed using UM-SCC-047 (**A**) or UM-SCC-22B (**B**) cells using transient transfection. The image of cells that had invaded through matrigel (Supplementary Figure 5) was processed and quantified in Photoshop. Both UM-SCC-047 and UM-SCC-22B cells had similar 60% invasion when treated with control constructs (empty vector for ectopic expression or non-targeting siRNA pool for RNAi). The migration of each cell was dysregulated significantly by ectopic *DTX1* overexpression (*a*, UM-SCC-047 cells) or by transient *DTX1* downregulation (*b*, UM-SCC-22B cells). *P-value* were calculated by *t-test* for experiments performed in triplicate. Transfection efficiency for each experiment was confirmed by qRT-PCR (Supplementary Figure 4).

Since *DTX1* was downregulated in HNSCC tumor samples, (Figures 2, 3, 4) it is expected to have tumor-suppressor properties. Overexpression and silencing of *DTX1* expression *in vitro* did not affect cell proliferation (3 immortalized normal keratinocyte and 6 HNSCC cell lines were tested, data not shown). On the other hand, recent data suggest that *DTX1* may play a role in inhibition of invasion in osteosarcoma [33]. In order to evaluate if *DTX1* could modify the invasiveness of HNSCC cells as well, we performed matrigel cell migration assays. UM-SCC-047 and UM-SCC-22B were selected due to their increased mobility relative to other HNSCC cell lines necessary for invasion assay. Base-line *DTX1* expression analysis determined that UM-SCC-047 had relatively lower gene expression and, therefore, was used for ectopic *DTX1* expression (Supplementary Figure 4A). On the other hand, UM-SCC-22B had a higher *DTX1* expression rate and, therefore, was used for knock-down RNAi experiments. Upregulation of *DTX1* via ectopic expression leaded to significant decrease of UM-SCC-047 cell invasion (*p* = 0.014, Figure 5A and Supplementary Figure 5A), while downregulation of *DTX1* by RNAi leaded to strong enhancement of cell invasiveness in UM-SCC-22B cell line (*p* = 0.004, Figure 5B and Supplementary Figure Supplementary 5B).

## DISCUSSION

Five year survival for HNSCC is only 50%, and there is a clear need for identification of novel cancer-specific therapeutic targets. This study integrated DNA methylation and gene expression to define cancer-related alterations. We performed genomic analysis with thorough confirmatory and functional validation, which identified DTX1 as a potential regulator of migration in HNSCC.

The integrated analysis and outlier statistics allowed us to correlate gene expression with promoter methylation and discriminate candidates that were biologically relevant. Indeed, all 15 high-value candidates identified through these methods (Table 1) have been described as cancer drivers in other cancer types. Thus, *DTX1* is a regulator of the NOTCH pathway; this pathway was recently found both downregulated in HNSCC via *NOTCH1* mutations and upregulated in HNSCC via amplifications of NOTCH's ligands and receptors [12–14, 22]. *DTX1* is upregulated in thymic tumors and in glioblastoma [46, 47], and it inhibits osteoblastoma cell invasion [33]. Moreover, multiple *DTX1* polymorphisms were found in non-small cell lung and B-cell precursor acute lymphoblastic leukemia patients [48, 49], which most likely associate with inactivation of *DTX1* and further NOTCH activation in these cancers. *BANK1* is downregulated in lymphoma and colorectal cancers [30, 32]. Overexpressed *BIN2* fusions were detected in myeloproliferative neoplasms [31]. Out of other candidates, *ATP2A3* has been shown to be mutated and downregulated in HNSCC, lung, colon and central nervous system cancers [50]. Several polymorphism of *CYP1B1* are found in many cancer types, including tobacco-related cancers; *CYP1B1* is also overexpressed in ovarian cancer [51, 52]. Wnt-pathway receptor *FZD3* is strongly expressed in colorectal and non-melanoma skin cancer and during chronic lymphocytic leukemia, CLL [53–55]. *ORAOV1* is an overexpressed marker of oral SCC [56]. Information about other genes can be found in Table 1.

In order to confirm the role of identified candidate genes, the 3 candidates were independently validated in multiple cohorts. As acknowledged by Mirghani et al [57], high throughput data is often poorly validated, with biases inherent to a single institution cohort or single methodology. To improve the candidate discovery pipeline, we employed both single-institution and multi-institutional HNSCC cohorts, and utilized diverse detection platforms: Illumina exon array, RNA-Seq and qRT-PCR for gene expression; and Methylation arrays 27 and 450, as well as bisulfite sequencing for DNA methylation. Moreover the 3 genes were confirmed to be hypomethylated and upregulated in healthy tissues of non-cancer patients and in non-cancer tumor-adjacent tissues of HNSCC patients with minor exclusions. While only 3 exemplary candidate genes out of the total 15 candidates were evaluated, the others may be expected to have strong differential expression and methylation in HNSCC regardless of sample cohort and detection tool.

Analysis of gene expression of NOTCH pathway members suggested that *DTX1* downregulation in HNSCC is correlated with downregulation of some NOTCH pathway genes including *DTX3*, *DLL3*, and *MFNG*, as well as genes downstream of NOTCH (*NEUROG3* and *GATA4*). Furthermore, *DTX1* expression was inversely correlated with *HES1* expression. These results confirm prior data that *HES1* is a negative regulator of *NEUROG3* and *DTX*s expression [33, 45]. DTX1 carries a putative SH3-binding domain and binds to the intracellular domain of NOTCH (ICN) [42]. One mechanism by which DTX1 may negatively regulate NOTCH is through ubiquitination; DTX1 may be an E3 ubiquitin ligase, as it contains a RING finger and two WWE domains [58]. Zhang and colleagues [33] proposed that DTX1 may bind and ubiqutiniate NOTCH's ICN, leading to negative regulation of NOTCH pathway, which was consistent with our findings (Supplementary Figure S6). HEY1 also negatively regulates *GATA4* [44], which was downregulated in parallel with *DTX1*.

Overall, *DTX1* was downregulated in the entire HNSCC discovery cohort, together with 20 other NOTCH pathway genes including *DLLs*, *NOTCH1*, *NOTCH2* and *NOTCH4* (Supplementary Table 4). *JAG1*, *JAG2*, *HEY1* and *HES1* had relatively higher expression in tumor samples compared to the pool of normal controls (Supplementary Table 4). This data suggested that *DTX1* expression can serve as biomarker of NOTCH pathway inhibition. While the NOTCH pathway is activated in many cancer types, *DTX1* is seen to be downregulated in osteoblastoma and HNSCC, while it is upregulated in glioblastoma [22, 33, 46, 59]. In addition, the NOTCH pathway was also found to be downregulated in thyroid cancer and in subgroup of HNSCC patients [22, 60]. Notably, genetic alterations of *NOTCH1* were recently found to dysregulate the NOTCH pathway in HNSCC [12, 13]. However, we did not find any correlation of *DTX1* expression with presence of *NOTCH1* mutations [14, 22, 61, 62]. Interestingly, 1/3 of NOTCH pathway genes are co-downregulated with *DTX1* (such as *DLLs*, *DTXs*, and *NOTCH4*), while the rest of the NOTCH pathway genes were upregulated in the low *DTX1* expression subgroup (genes including *NOTCH1-3*, *HES1*, *HEY1*, and *JAG1*, Supplementary Tables 4, 5). These results correlate with recent discovery that NOTCH pathway has complex gene interactions and dual function, where it is activated in some tumors while inactivated in others [22, 61]. Notably, there were more HPV-related (HPV+) patients in the lower *DTX1* expression group (*n* = 9 HPV+ patients), compared to the higher *DTX1* expression group (*n* = 4 HPV+ patients, Fisher's exact test *p-value* = 0.185). There were 4 times more oral cavity patients in the higher *DTX1* expression group (*n* = 8, 0.069). Unfortunately, those or other correlations with clinical characteristics did not reach statistical significance. This is the first report that has demonstrated that DTX1 blocks HNSCC migration, which is in agreement with recently published data suggesting that DTX1 blocks osteosarcoma invasiveness [33]. Results of current analysis suggests that in HNSCC, downregulation of *DTX1* by DNA methylation leads to more aggressive behavior of HNSCC cells.

Since the expression of *NEUROG3* and *GATA4* (downstream of HES1 and HEY1 respectively) was downregulated in parallel with *DTX1*, we speculate, that *NEUROG3* and *GATA4* have a negative effect on cell migration (Supplementary Figure 6). The molecular mechanism by which DTX1 blocks HNSCC cell migration needs to be further evaluated. Additional analysis of role of *DTX1* on cell proliferation did not show any significant changes of cell growth depending of *DTX1* expression (data not shown).

We have to acknowledge several limitations of our study: 1) clinical characteristics between tumor and non-tumor groups do not match in patients in both discovery and validation cohorts (Supplementary Table 1), due to peculiarity of UPPP and HNSCC populations [21–24]. Nonetheless the employed UPPP population helped revealing strong cancer-specific signatures of HNSCC in previous studies [21–24]. Moreover, employment of TCGA's control population with matched clinical characteristics confirmed our original discovery of hypermethylation and downregulation of candidate genes, especially the leading candidate, *DTX1*, in tumor samples. 2) Utilization of older generation DNA methylation array data (Illumina Infinium HumanMethylation27 BeadChip) narrowed down the list of candidates by lack of available promoter-methylation data for several genes. Nonetheless this is one of the largest HNSCC cohorts, after the employed TCGA, with publicly available matched gene expression and promoter methylation data [21–24]. We see this study as confirmation of our pipeline for discovery of biologically-relevant candidates and smaller number of candidate genes helped us focus only on the limited number of genes within a limited time frame. 3) Not all candidates were functionally evaluated within given time frame, but will be used for further independent analyses. The complete list of high priority candidates discovered during this project will become available for the research community for their prospective studies.

Thousands of alterations can be detected by different independent high throughput platforms, given their high sensitivity. Integration of gene expression and DNA methylation high throughput data focused study to a limited list of relevant genes with potential roles in HNSCC carcinogenesis. Employment of well-considered statistical approaches, cross cohort validation, and complementary detection tools allowed us to discover an epigenetically regulated tumor suppressor gene, *DTX1*, which controls HNSCC cell migration.

## MATERIALS AND METHODS

### Specimen cohort assembly

We used two independent cohorts of specimens, each composed of primary head and neck squamous cell carcinoma (HNSCC) tissue specimens and control specimens comprising normal mucosal samples from uvulopalatopharyngoplasty (UPPP) surgeries of non-cancer affected patients. The discovery cohort comprised of 44 HNSCC and 25 normal UPPP samples, as described in previous publications [22, 23]. The validation cohort comprised of 61 HNSCC and 28 normal UPPP samples, is reported for the first time. The demographic and clinico-pathological characteristics of patients from the discovery and validation cohorts are listed in Supplementary Table 1. The clinical differences between tumor and control populations in the discovery cohort were previously identified and acknowledged [21–24].

All tissue samples were obtained from the Johns Hopkins Tissue Core, as a part of the Head and Neck Cancer Specialized Program of Research Excellence (HNC-SPORE). These samples were acquired under Internal Review Board-approved research protocol #NA_00036235. Informed consent was obtained from all patients recruited under this protocol prior to participation in the study.

### Tissue processing

Two Johns Hopkins Hospital Pathologists (WHW and JAB) independently confirmed that all primary tumor samples were consistent with HNSCC. After this, all tumor tissues were microdissected to yield at least 80% tumor purity. All tissue specimens were stored at −140°C until a cut and extraction were performed. For each extraction, a 0.35 mm thick cut of tissue was used.

### RNA preparation

RNA was isolated from 0.35 mm thick frozen tissue cuts with the mirVana miRNA Isolation Kit (Ambion, Forster City, CA) at room temperature as per manufacturer's recommendations. The concentration of the isolated RNA was quantified using the NanoDrop spectrophotometer (Thermo Fisher Scientific, Waltham, MA).

### DNA preparation

Similarly, 0.35 mm thick frozen tissue cuts were digested in 1% SDS (Sigma-Aldrich, St. Louis, MO) and 50 μg/ml proteinase K (Invitrogen, Carlsbad, CA) solution at 48°C for 48 hours. The DNA was purified by phenol-chloroform extraction and ethanol precipitation as previously described [63]. DNA was resuspended in LoTE buffer, and the DNA concentration was quantified using the NanoDrop spectrophotometer.

### High throughput transcriptional and methylation profiling data

High throughput data of gene expression and DNA methylation was obtained from the discovery cohort which was previously published using the methods described previously [22, 23]. The gene expression data for the discovery cohort was obtained from Affymetrix HuEx1.0 GeneChips (containing 1.4 million probes) and is available on the NCBI Gene Expression Omnibus (GEO) public repository (GEO33205). DNA methylation data for the discovery cohort was obtained using Illumina Infinium HumanMethylation27 BeadChip (probing 27,578 CpG dinucleotides) is also publically available (GEO33202). The data from both platforms can be downloaded from the combined superSeries GSE33232.

### Reverse transcription and quantitative real time PCR

Validation of gene expression was performed in the independent validation cohort using reverse transcription and quantitative real-time PCR. One microgram of RNA from each sample in the validation cohort was reverse transcribed using the High Capacity cDNA Reverse Transcription Kit (Applied Biosystems, Forster City, CA). Quantitative real-time PCR (qRT-PCR) was performed using gene-specific expression assays (Supplementary Table 3) and Universal PCR Master Mix on a 7900HT real-time PCR machine (Applied Biosystems) as per manufacturer's recommendations. Expression of the gene of interest was quantified in triplicate relative to *GAPDH* expression using the 2-ΔΔCT method [64]. We also confirmed that *GAPDH* expression was not significantly different in normal and HNSCC samples.

### Bisulfite treatment and bisulfite genomic sequencing

Validation of DNA methylation was performed in the validation cohort through bisulfite genomic sequencing. The EpiTect Bisulfite Kit (Qiagen, Valencia, CA) was used to convert unmethylated cytosines to uracil in genomic DNA. Touch-down PCR was performed on bisulfite-converted DNA with primers designed to span areas of CpG islands for each gene promoter (Supplementary Table 3). −[65]. The PCR products were purified using the QIAquick 96 PCR Purification Kit (Qiagen). The purified PCR product from bisulfite-converted DNA for each sample and gene was sequenced (Genewiz, South Plainfield, NJ). Relative heights of C and T peaks measured on sequencing were then used to assign the DNA methylation status as unmethylated, methylated or hemimethylated.

### TCGA sample selection

The TCGA project for HNSCC was recently completed and published [39]. Overall, the analysis included *n* = 279 HNSCC samples. Such a cohort was different from the employed Johns Hopkins cohorts by increased number of oral cavity samples, samples with TNM stage II–III, and decreased HPV-positive samples. In order to “match” clinical characteristics of the analyzed TCGA cohort, we removed oral cavity samples with TNM stage II and III. We did not do any additional manipulation of the TCGA cohort to keep the “matching” procedure simple and randomized, as well as to avoid any potential biases due to non-random sample selection. Since oral cavity samples are predominately HPV-negative, this helped to increase relative percentage of HPV-positive HNSCC samples within the new “matched” TCGA population (*n* = 222 total). Notably, the new “matched” TCGA cohort did not significantly change other clinical characteristics that were relevant to the Johns Hopkins cohort. This “matched” TCGA cohort (Supplementary Table 1) was used for validation purposes (Figure 4).

### Cell culture

### Cell lines and cell culture conditions

Human HNSCC cell lines UM-SCC-047 and UM-SCC-22B were provided by Dr. Thomas Carey (University of Michigan) for the functional experiments. Each cell line was authenticated using a Short Tandem Repeat (STR) Identifiler kit (Applied Biosystems). Cells were grown on high-glucose DMEM media (Clontech, Mountain View, CA), supplemented by 10% fetal bovine serum (FBS) and 1% Penicillin-Streptomycin at 37°C in 5% CO_2_.

### Transient transfection

For knockdown assays, the expression of *DTX1* gene was downregulated by ON-TARGETplus siRNA SMARTpool RNA (L-006525-00-0005, Thermo Scientific, Waltham, MA) using RNAiMAX transfection reagent (Life Technologies, Carlsbad, CA). Non-targeting SMARTpool RNA (D-001810-10-05, Life Technologies) was used as a control. The transfection efficiency was confirmed by qRT-PCR.

The ectopic overexpression of *DTX1* was achieved with pCMV6-Entry-DTX1 plasmid (RC208338, Origene, Rockville, MD) using FuGENE Extreme 9 transfection reagent (Roche, Nutley, NJ). Empty pCMV6-Entry (PS100001, Origene) was used as a control. Transfection efficiency was confirmed by DTX1-specific qRT-PCR using the same qRT-PCR technique as described earlier for tissue RNA analysis.

### Matrigel invasion assay

We performed the Matrigel invasion assay to assess the migration and invasion ability of transfected cells with over expression and under expression of *DTX1* using techniques described in previous publications [66]. In short, 8-μm pore filter inserts in 24-well plates (Sigma-Aldrich) coated with Matrigel (BD Biosciences, San Jose, CA) was used. Cells were transfected for 24 hours and then were trypsinized, washed three times with serum-free DMEM media and resuspended in serum-free DMEM to obtain the concentration of 10^6^ cells/ml. An aliquot of 100 μl of cells were plated onto each insert. Chemo-attractant media with 10% FBS (600 μl) was added to the bottom of a 24-well plate. Each insert, with cell suspensions, was placed into the individual well with chemo-attractant media. After 24 hours of incubation at 37°C in 5% CO_2_, the inserts were removed from the media. Cells on the upper surface of the insert that did not invade through the membrane were removed with a cotton swab. The cells that had migrated to the lower surface of the membrane, facing the chemo-attractant media, were fixed by 10% formaldehyde and stained by 1% crystal violet. The membranes with fixed and stained cells were removed, mounted onto slides and photographed by microscopy at 4× magnification. Each experiment was performed in triplicate.

### Matrigel migration quantification

The 4× magnified images of the insert membrane were analyzed using Adobe Photoshop SC6 (Adobe Systems, McLean, VA). Stained cell-occupied image area (purple) was selected by the “Color Range” tool with 70% fuzziness. The number of pixels in the entire image and the number of pixels within areas occupied by cells were calculated by the “Histogram” tool of Photoshop. The percentage of image field occupied by cells was calculated as total number of pixels occupied by cells relative to the total number of pixels. Triplicate images were analyzed for each experiment and the mean of percentage of the cell-occupied image field was calculated.

### Statistical analysis

### Expression array normalization

The gene expression data from GEO33205 used Robust Multiarray Average (RMA) implemented in the Bioconductor oligo package [67, 68] for normalization, as previously described [21, 22]. The gene level expression estimates were calculated as the mean expression levels among all core probes linked to the same gene, yielding 22,011 genes.

### DNA methylation array normalization

For promoter methylation data available at GEO33202, beta values (percent methylation) were estimated from unmethylated (U) and methylated (M) measurements on a probe level basis: β = M/(M+U) [23, 24]. The gene level estimates were produced by choosing the highest methylation levels among all probes linked to the same gene yielding, 14,477 individual genes.

### Outlier analysis

HNSCC is a heterogeneous disease with cancer-related changes detected in only a small portion of the samples [12–14, 39]. Unfortunately, such changes are poorly detected by conventional statistical approaches such as the *t-test*. A standard method employed in cancer research for outlier analysis is Cancer Outlier Profile Analysis (COPA) and its derivatives [28, 69], which generate statistics by comparing the outlier distributions to an empirical null generated by permutation of class labels. However, these methods have limitations when counting outliers since the distribution of medians and median absolute deviations permits outliers to be called in cases where the deviations are biologically insignificant. Therefore, we recently implemented a rank sum outlier approach, modified from Ghosh [70], where a minimum change levels was set for the calling of an outlier [20]. Such methods allowed us to eliminate many outliers where change is not biologically meaningful (e.g., a gene expression change of less than 10%, or 2.35 log fold change, between any two samples). The outlier statistics was used exclusively for discovery purposes.

To discover the genes with changes in expression, we applied the outlier statistics described above in reference [20] to the array gene expression data set, containing the 22,011 genes for each of 44 HNSCC tissue samples from the discovery cohort. The signals from 25 normal samples from the same cohort were used to establish the empirical null level for each gene. We calculated outlier score for both left-tail (10th percentile) and right-tail (90th percentile) cases, which allowed us to define outliers that were downregulated and upregulated, respectively [20, 70]. The outlier statistics yielded an outlier score, which quantified the number of tumors with gene expression values that were outliers from the distribution defined by normal as defined in previously published work [20]. Each of the 22,011 genes was assigned its outlier score and ranked from the largest to the smallest. Outlier analysis does not have a cut-off value of significance, such as 0.05 for *p-value*; therefore for the convenience we manually selected the genes with the top 76 outlier scores as candidates (manual outlier score cut-off set = 2.3, Supplementary Table 2). All outlier analyses were performed with custom scripts in R adapted from [20].

### The pathway enrichment analysis

The pathway enrichment analysis utilized gene expression data was used for 43 NOTCH pathway genes (KEGG database and [22] without *DTX1*) with available Affymetrix Exon Array data. Differential expression analysis of NOTCH pathway and downstream genes was performed with empirical Bayes moderated t-statistics using limma R package 2.12.0 [71]. Contrasts were formulated to define the difference between different gene expression in groups of patient samples: tumor samples with higher *DTX1* expression, tumor samples with lower *DTX1* expression, and non-cancer samples. *P*-values for differential expression statistics were reported after FDR adjustment with Benjamini-Hotchberg correction [72] among 43 NOTCH pathway and 9 downstream genes, including *DTX1* in both sets. Genes with FDR adjusted *p*-values below 0.05 were called significantly differentially expressed. Pathway-level statistics were computed by applying the limma function geneSetTest to the empirical Bayes moderated t-statistics for each contrast with the alternative hypothesis of “either” specifying that genes in the set are up or down regulated as a group.

### Expression-methylation correlation

We utilized a correlation analysis to associate changes in gene expression with epigenetic regulation. Specifically, we computed Spearman correlation coefficients between gene-level estimates of DNA methylation and expression for each candidate gene inferred from outlier statistics. Candidate genes with negative correlations between expression and methylation were selected. All correlation analyses were performed using limma R package 2.12.0 [71].

### *P*-value calculation

Log transform of gene expression values from array, normalized qRT-PCR gene expression values, DNA methylation β-values, and image field percentage values were compared for HNSCC and control samples using the Student *t-test*. We used *t-test* for all our validation step as it is more stringent than outlier test, used exclusively for discovery purposes. Bisulfite sequencing results were compared for HNSCC and control samples using the two-tailed Fisher exact test.

## SUPPLEMENTARY MATERIALS FIGURES AND TABLES












