# Supplementary material for: Integrative computational analysis of transcriptional and epigenetic alterations implicates DTX1 as a putative tumor suppressor gene in HNSCC
Source: Oncotarget. 2017 Jan 27;8(9):15349–63. doi: 10.18632/oncotarget.14856 (PMC5362490; doi:10.18632/oncotarget.14856)
Supplement: Supplementary file 1 [file oncotarget-08-15349-s001.pdf]

Integrative computational analysis of transcriptional and epigenetic alterations implicates *DTX1* as a putative tumor suppressor gene in HNSCC

Supplementary Materials

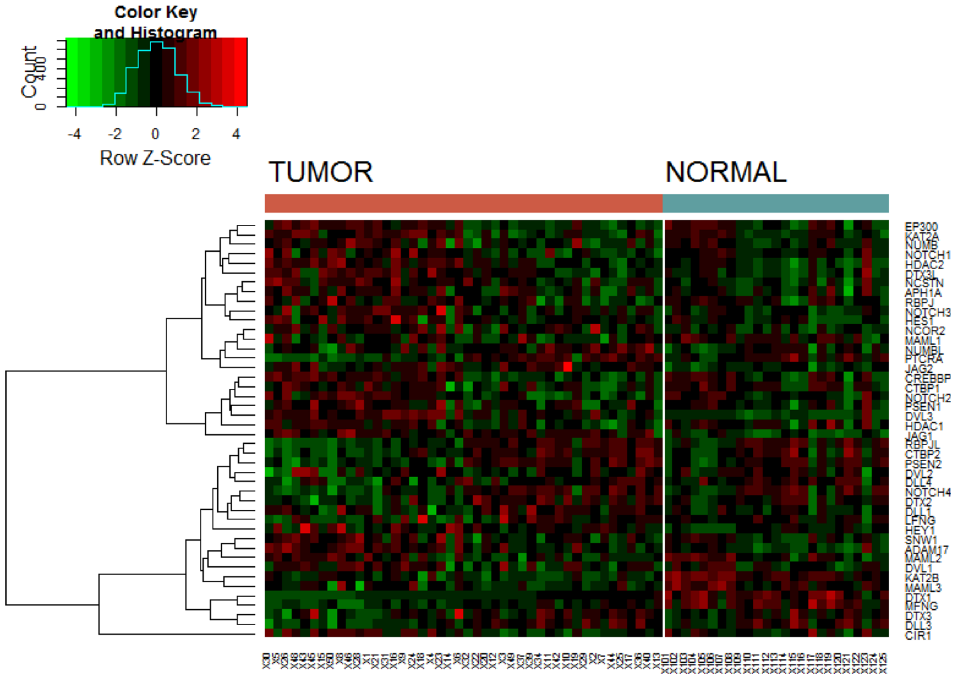

Supplementary Figure 1: Heat map of gene expression of NOTCH signaling pathway genes in the discovery cohort. Gene expression was assessed by Affymetrix HuEx 1.0 GeneChip platform in the discovery cohort of 44 HNSCC tumors and 25 normal tissues. All HNSCC tumor samples are labeled salmon and normal tissues are blue. NOTCH pathway genes (n = 44) were from KEGG database and Reference #21. Genes in the heat map are shown in rows; each individual sample is shown in one column. This scaled expression value, denoted as the row Z-score, is plotted in green-red color scale with green indicating low expression and red indicating high expression. Tumor samples are sorted by *DTX1* gene expression. Unsupervised hierarchical clustering of genes and samples was based on Pearson's correlation.

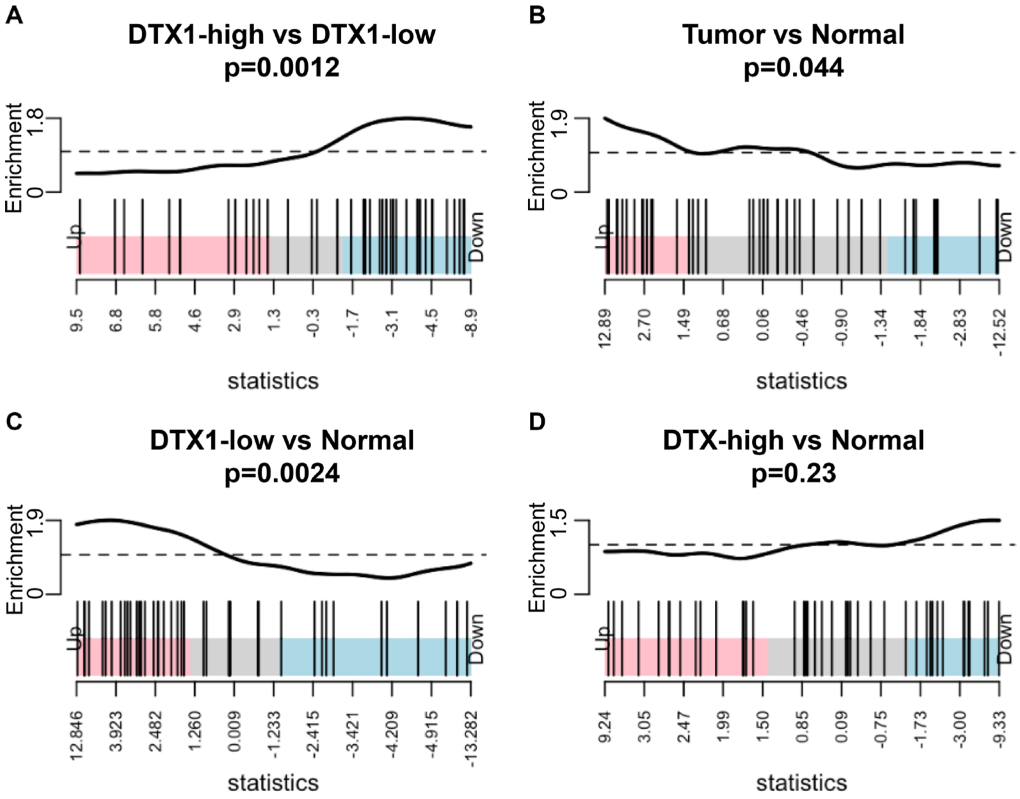

Supplementary Figure 2: NOTCH pathway enrichment analysis. We have performed limma pathway enrichment analysis to define the difference between different group of patient samples: (**A)** Tumor samples with higher *DTX1* expression vs tumor samples with lower *DTX1* expression; (**B**) All tumor samples vs all normal samples; (**C**) Tumor samples with lower *DTX1* expression vs all normal samples; (**D**) Tumor samples with higher *DTX1* expression vs all normal samples. The FDR adjusted *p*-values were calculated without accounting for *DTX1* expression to eliminate the biases.

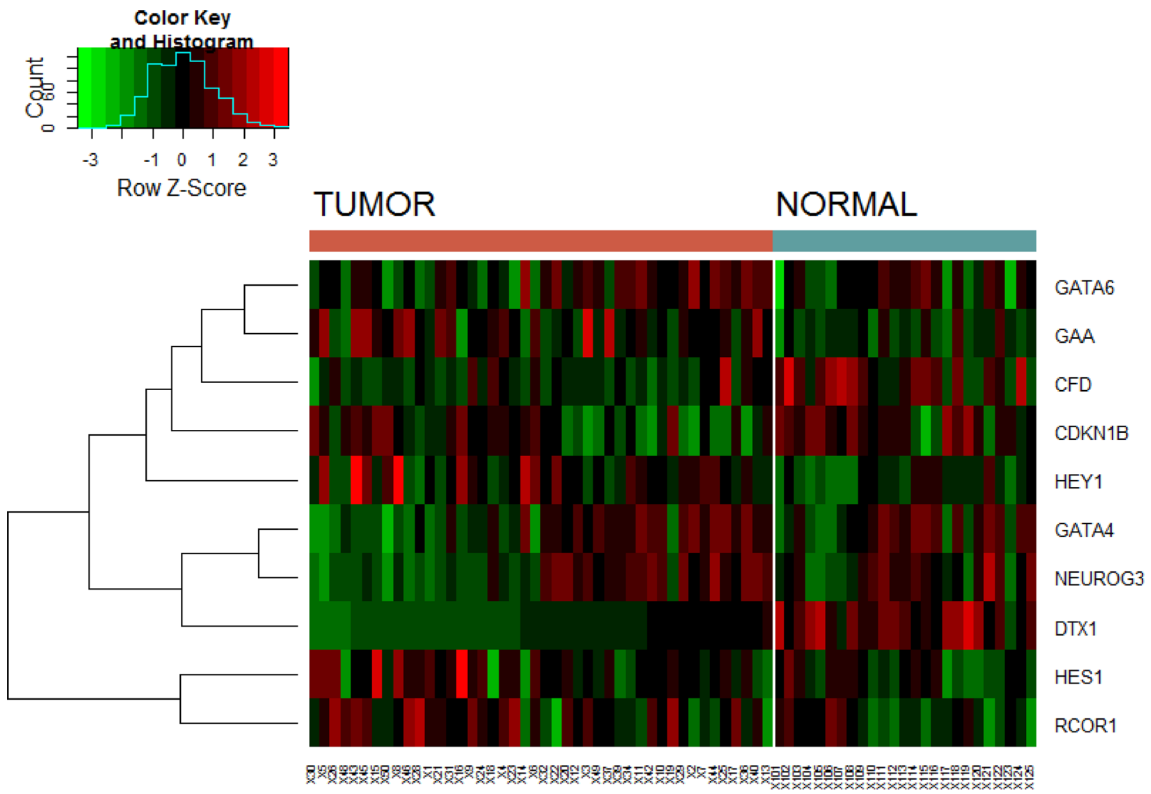

Supplementary Figure 3: Expression analysis of the genes downstream of NOTCH signaling pathway in the discovery cohort. Gene expression was assessed by Affymetrix HuEx 1.0 GeneChip platform in the discovery cohort of 44 HNSCC tumors and 25 normal tissues. As proposed by literature, *HES1* and *HEY1* genes are downstream target of NOTCH pathway, where expression of *NEUROG3*, *GAA*, *CDKN18*, *RCOR1*, and *CFD* are regulated by HES1; expression of *GATA4* and *GATA6* are regulated by *HEY1*. Thus, these genes together with *DTX1* were used to build the heat-map. All HNSCC tumor samples are labeled salmon and normal tissues are blue. Genes in heat map are shown in rows; each individual sample is shown in one column. This scaled expression value, denoted as the row Z-score, is plotted in green-red color scale with green indicating low expression and red indicating high expression.

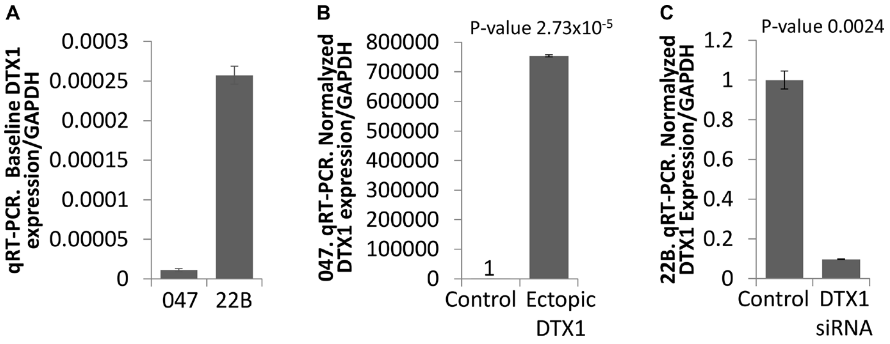

Supplementary Figure 4: Transfection efficiency. (**A**) The baseline expression of *DTX1* in UM-SCC-047 (047) and UM-SCC-22B (22B). The RNA level of DTX1 was evaluated during control and experiments with ectopic *DTX1* (UM-SCC-047, (**B**) expression or DTX1 RNAi (UM-SCC-22B, (**C**). Values were normalized to *GAPDH* expression and scaled to 1 (in B and C) in the control experiment. *P*-value were calculated using *t*-test for three repeats. Values are mean ± SE.

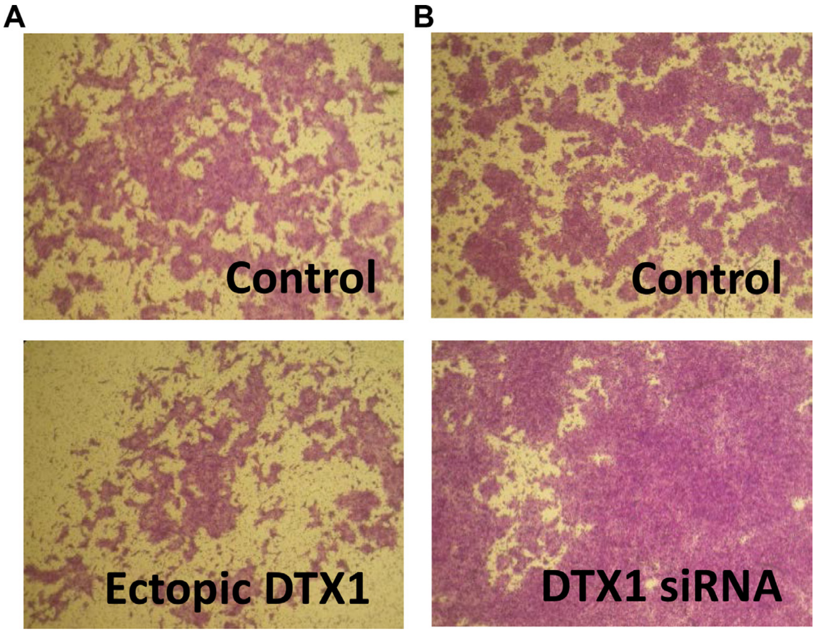

Supplementary Figure 5: DTX1 blocks HNSCC invasiveness. Migration assay was performed using UM-SCC-047 (**A**) or UM-SCC-22B (**B**) cells using transient transfection described in methods. The image of cells that invaded through the matrigel (A and B) during control treatment with empty vector (A, top) or non-targeting siRNA pool (B, top), or with *DTX1*-expressing plasmid (A, bottom), or *DTX1* siRNA pool (B, bottom). The experiments were performed in triplicate and the representative images are shown. Transfection efficiency was confirmed by qRT-PCR (Supplementary Figure 4).

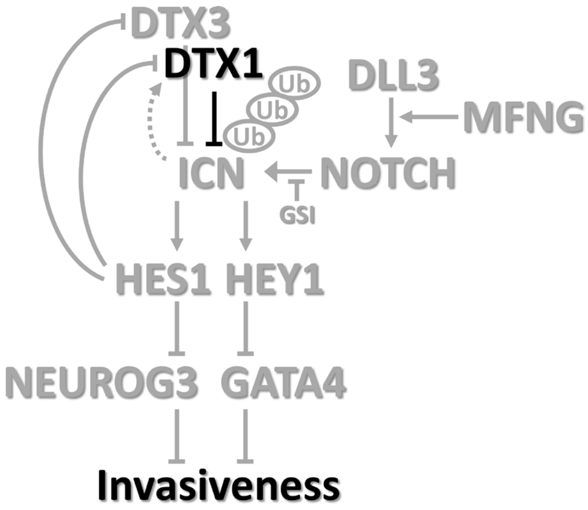

Supplementary Figure 6: The proposed role of DTX1 on NOTCH pathway and cell invasiveness.

Supplementary Table 1: Clinical data for the discover, validation and TCGA cohorts. See Supplementary_Table_1

Supplementary Table 2: Outlier score and Spearman coefficient for 76 candidates from the expression. See Supplementary_Table_2

Supplementary Table 3: Bisulfite sequencing primers and expression assays used in the study. See Supplementary_Table_3

Supplementary Table 4: NOTCH pathway genes mean values and FDR adjusted *p*-values for different patient groups within the discovery cohort. See Supplementary_Table_4

Supplementary Table 5: NOTCH pathway downstream genes mean values and FDR adjusted *p*-values for different patient groups within the discovery cohort. See Supplementary_Table_5
